# Comparing the hemodynamic effects of six different colloids

**DOI:** 10.1186/cc12315

**Published:** 2013-03-19

**Authors:** N Kteniadakis, V Grosomanidis, E Oloktsidou, B Fyntanidou, C Nouris, K Kotzampasi, D Vasilakos

**Affiliations:** 1 Aristotle Medical School, Thessaloniki, Greece

## Introduction

Despite the fact that colloids are widely used, doubt still remains as to which colloid is the best and what is the ideal volume of administration. The aim of our study was to compare the hemodynamic effects of six different colloids.

## Methods

A total of 180 patients were enrolled in our study undergoing general surgery procedures, who were assigned to six groups (A, B, C, D, E, F). After fasting fluid replacement and induction of anesthesia, 500 ml Gelofusine, Haemaccel, Voluven, HES 6%, HES 10% and Hemohes were administered to each group respectively. An oesophageal Doppler monitor probe was inserted into the patients for measuring stroke volume (SV) and cardiac output (CO). Standard monitoring included ECG, IBP, ETCO_2 _and SpO_2_, and heart rate (HR). Systemic arterial pressure (SAP), SV and CO were recorded directly before the administration of any colloid (T0) and every 5 minutes for the next 1 hour (T1 to T12). Kolmogorov-Smirnov was used to test normal distribution of data and ANOVA was used for the statistical analysis. *P <*0.05 was considered statistically significant.

## Results

Demographic data and ASA classification did not differ statistically significant among the six groups of the study. CO, SV, HR and SAP did not show any statistically significant evolution compared with their baseline value during the study period. Moreover, there were no statistically significant differences among the six study groups with regard to any of the recorded parameters.

## Conclusion

According to our results, volume replacement with the six colloids tested in our study did not result in any hemodynamic response. Within comparison of these six colloids did not reveal any statistically significant difference in any of the parameters recorded according to our protocol.

